# The quinter­nary thio­phosphate Cs_0.5_Ag_0.5_Nb_2_PS_10_

**DOI:** 10.1107/S1600536810021768

**Published:** 2010-06-16

**Authors:** Sojeong Park, Hoseop Yun

**Affiliations:** a Division of Energy Systems Research and Department of Chemistry, Ajou University, Suwon 443-749, Republic of Korea

## Abstract

The quinter­nary thio­phosphate Cs_0.5_Ag_0.5_Nb_2_PS_10_, cesium silver tris­(disulfido)[tetra­thio­phosphato(V)]diniobate(IV), has been prepared from the elements using a CsCl flux. The crystal structure is made up of _∞_^1^[Nb_2_PS_10_] chains expanding along [010]. These chains are built up from bicapped trigonal-prismatic [Nb_2_S_12_] units and tetra­hedral [PS_4_] groups and are linked through a linear S—Ag—S bridge, forming a two-dimensional layer. These layers then stack on top of each other, completing the three-dimensional structure with an undulating van der Waals gap. The disordered Cs^+^ ions reside on sites with half-occupation in the voids of this arrangement. Short [2.8843 (5) Å] and long [3.7316 (4) Å] Nb—Nb distances alternate along the chains, and anionic S_2_^2−^ and S^2−^ species are observed. The charge balance of the com­pound can be represented by the formula [Cs^+^]_0.5_[Ag^+^]_0.5_[Nb^4+^]_2_[PS_4_^3−^][S_2_^2−^]_3_.

## Related literature

For Nb_2_PS_10_-related quaternary thio­phosphates, see: Do & Yun (1996 ▶) for KNb_2_PS_10_, Kim & Yun (2002 ▶) for RbNb_2_PS_10_, Kwak *et al.* (2007 ▶) for CsNb_2_PS_10_, Bang *et al.* (2008 ▶) for TlNb_2_PS_10_, and Do & Yun (2009 ▶) for Ag_0.88_Nb_2_PS_10_. For quintenary thio­phosphates, see: Kwak & Yun (2008 ▶) for K_0.34_Cu_0.5_Nb_2_PS_10_, Dong *et al.* (2005*a* ▶) for K_0.5_Ag_0.5_Nb_2_PS_10_, and Dong *et al.* (2005*b* ▶) for Rb_0.38_Ag_0.5_Nb_2_PS_10_. *PLATON* (Spek, 2009 ▶) was used for structure validation. For typical Nb—P and P—S bond length, see: Brec *et al.* (1983 ▶), and for typical Nb^4+^–Nb^4+^ bond lengths, see: Angenault *et al.* (2000 ▶). For general background, see: Lee *et al.* (1988 ▶).

## Experimental

### 

#### Crystal data

Cs_0.5_Ag_0.5_Nb_2_PS_10_
*M* *_r_* = 657.78
Monoclinic, 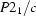

*a* = 7.3594 (3) Å
*b* = 12.8534 (4) Å
*c* = 13.7788 (6) Å
β = 91.0886 (12)°
*V* = 1303.15 (8) Å^3^
*Z* = 4
Mo *K*α radiation
μ = 5.54 mm^−1^
*T* = 290 K
0.30 × 0.06 × 0.04 mm

#### Data collection

Rigaku R-AXIS RAPID diffractometer
Absorption correction: multi-scan (*ABSCOR*; Higashi, 1995 ▶) *T*_min_ = 0.602, *T*_max_ = 1.000
12389 measured reflections
2991 independent reflections
2430 reflections with *I* > 2σ(*I*)
*R*_int_ = 0.049

#### Refinement

*R*[*F* ^2^ > 2σ(*F* ^2^)] = 0.034
*wR*(*F* ^2^) = 0.075
*S* = 1.08
2991 reflections
133 parameters
Δρ_max_ = 1.18 e Å^−3^
Δρ_min_ = −1.27 e Å^−3^

### 

Data collection: *RAPID-AUTO* (Rigaku, 2006 ▶); cell refinement: *RAPID-AUTO*; data reduction: *RAPID-AUTO*; program(s) used to solve structure: *SHELXS97* (Sheldrick, 2008 ▶); program(s) used to refine structure: *SHELXL97* (Sheldrick, 2008 ▶); molecular graphics: locally modified version of *ORTEP* (Johnson, 1965 ▶); software used to prepare material for publication: *WinGX* (Farrugia, 1999 ▶).

## Supplementary Material

Crystal structure: contains datablocks global, I. DOI: 10.1107/S1600536810021768/wm2357sup1.cif

Structure factors: contains datablocks I. DOI: 10.1107/S1600536810021768/wm2357Isup2.hkl

Additional supplementary materials:  crystallographic information; 3D view; checkCIF report

## Figures and Tables

**Table:** 

Ag—S1	2.4625 (13)
Nb1—S4^i^	2.4953 (13)
Nb1—S7^i^	2.4958 (12)
Nb1—S8^i^	2.5055 (13)
Nb1—S10^i^	2.5231 (13)
Nb1—S9	2.5658 (12)
Nb1—S2	2.5895 (12)
Nb1—S5	2.5993 (13)
Nb1—S6	2.6103 (12)
Nb2—S10	2.4910 (13)
Nb2—S7	2.4932 (13)
Nb2—S4	2.4985 (12)
Nb2—S8	2.5075 (12)
Nb2—S5	2.5643 (13)
Nb2—S9	2.5670 (12)
Nb2—S3	2.5920 (12)
Nb2—S6	2.6250 (12)
P—S1	1.9962 (18)
P—S3	2.0391 (17)
P—S2	2.0527 (17)
P—S6	2.0851 (17)

**Table:** 

S1—Ag—S1^ii^	180

Symmetry codes: (i) 
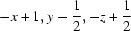
; (ii) 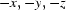.

**Fig. 1.**
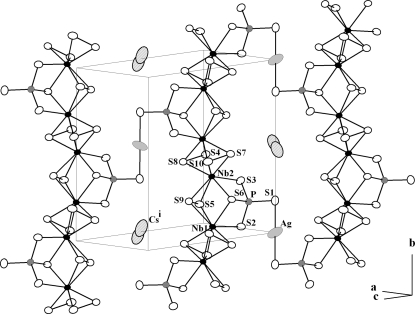
A view of the Cs0.5Ag0.5Nb2PS10 structure. Anisotropic displacement ellipsoids are drawn at the 90% probability level. Symmetry codes are given in Table 1.

## supplementary crystallographic information

### Comment

During an effort to expand representatives of group 5 transition metal thiophosphates by substituting various monovalent cations, we were able to prepare a new derivative in this system. Here we report the synthesis and characterization of the new layered quinternary thiophosphate, Cs_0.5_Ag_0.5_Nb_2_PS_10_.

The title compound is isostructural with the previously reported K_0.34_Cu_0.5_Nb_2_PS_10_ (Kwak & Yun, 2008). The _∞_^1^[Nb_2_PS_10_] chains found in this structure are composed of the typical biprismatic [Nb_2_S_12_] and tetrahedral [PS_4_] units. The Nb atoms are surrounded by 8 S atoms in a bicapped trigonal-prismatic fashion. Two prisms are sharing a rectangular face to form the [Nb_2_S_12_] unit. These units are bound through the S—S prism edges and through one of the capping sulfur atoms to make _∞_^1^[Nb_2_S_9_] chains. One of the S atoms at the prism edge and two other capping S atoms are bound to the P atom to which an additional S atom (S1) is attached to complete the _∞_^1^[Nb_2_PS_10_] chains. These anionic chains propagate parallel to [010] and are linked through the linear S—Ag—S bridge to form a two-dimensional layer along (201). These layers then stack on top of each other to complete the three-dimensional structure with an undulating van der Waals gap. The disordered Cs^+^ cations reside in the voids of this arrangement.

The Nb—S and P—S distances are in agreement with those found in other related phases (Brec *et al.*, 1983). Along the chain, The Nb(1)···Nb(2) interactions alternate in the sequence of one short (2.8843 (5) Å) and one long (3.7316 (4) Å) distance. The short distance is close to that of the typical Nb^4+^—Nb^4+^ bond (Angenault *et al.*, 2000), and the long Nb···Nb distance shows that there is no significant intermetallic bonding interaction. Such an arrangement is consistent with the high electric resistivity of the crystal along the needle axis (*b* axis).

The coordination around the Ag atom (1 symmetry) can be described as a [2 + 4] interaction. Four S atoms are bound to the Ag atoms in the plane (Ag—S6, 3.139 (3) Å, Ag—S9, 3.232 (3) Å), whereas two *trans* S atoms are coordinated to the Ag atom at short distances of Ag—S1 = 2.4625 (13) Å. The large ADPs of Ag could be explained by the second-order Jahn-Teller coupling between the filled Ag *e_g_* and the empty *s* orbitals (Lee *et al.*, 1988), which is a common trend of *d*^10^ elements. The charge balance of the compound can be represented by the formula [Cs^+^]_0.5_[Ag^+^]_0.5_[Nb^4+^]_2_[PS_4_^3-^][S_2_^2-^]_3_.

For Nb_2_PS_10_-related quaternary thiophosphates, see: Do & Yun (1996) for KNb_2_PS_10_, Kim & Yun (2002) for RbNb_2_PS_10_, Kwak *et al.* (2007) for CsNb_2_PS_10_, Bang *et al. *(2008) for TlNb_2_PS_10_, and Do & Yun (2009) for Ag_0.88_Nb_2_PS_10_; for quinternary thiophosphates, see: Kwak & Yun (2008) for K_0.34_Cu_0.5_Nb_2_PS_10_, Dong *et al.* (2005*a*) for K_0.5_Ag_0.5_Nb_2_PS_10_, and Dong *et al.* (2005*b*) for Rb_0.38_Ag_0.5_Nb_2_PS_10_.

### Experimental

Cs_0.5_Ag_0.5_Nb_2_PS_10_ was prepared by the reaction of elemental powders, using the reactive halide-flux technique. Ag powder (CERAC 99.999%), Nb powder (CERAC 99.8%), P powder (CERAC 99.5%) and S powder (Aldrich 99.999%) were mixed in a fused silica tube in a molar ratio of Ag:Nb:P:S=1:2:1:10 and then CsCl was added in a weight ratio of AgNb_2_PS_10_:CsCl=1:3. The tube was evacuated to 0.133 Pa, sealed and heated gradually (50 K/h) to 973 K, where it was kept for 72 h. The tube was cooled to room temperature at the rate of 4 K/h. The excess halide was removed with distilled water and dark red needle-shaped crystals were obtained. The crystals are stable in air and water. A qualitative X-ray fluorescence analysis of the needles indicated the presence of Cs, Ag, Nb, P, and S. The composition of the compound was determined by single-crystal X-ray diffraction.

### Refinement

Refinement went smoothly but the anisotropic displacement parameters (ADPs) of the Cs (Wyckoff position 4*e*) and Ag (2*a*) atoms were large compared with those of the other atoms. Because non-stoichiometry in these phases is sometimes observed and the distance between Cs atoms is too short if full occupancy is assumed, the occupancies of each metal atom were checked by refining the site occupation factors (SOFs) while those of the other atoms were fixed. With the non-stoichiometric model, the SOF of the Cs site was reduced significantly from 1 to 0.49 and the residuals improved also. As no evidence was found for ordering of the Cs site at Wyckoff position 2*c*, a statistically disordered structure was finally modelled. The final difference Fourier map showed that the highest residual electron density (1.18 e/Å^3^) is 0.94 Å from the Nb2 site and the deepest hole (-1.27 e/Å^3^) is 0.84 Å from the Nb2 site. No additional symmetry, as tested by *PLATON* (Spek, 2009), has been detected in this structure.

### Crystal data

**Table:**

Cs_0.5_Ag_0.5_Nb_2_PS_10_	*F*(000) = 1232
*M**_r_* = 657.78	*D*_x_ = 3.353 Mg m^−^^3^
Monoclinic, *P*2_1_/*c*	Mo *K*α radiation, λ = 0.71073 Å
Hall symbol: -P 2ybc	Cell parameters from 8832 reflections
*a* = 7.3594 (3) Å	θ = 3.2–27.5°
*b* = 12.8534 (4) Å	µ = 5.54 mm^−^^1^
*c* = 13.7788 (6) Å	*T* = 290 K
β = 91.0886 (12)°	Needle, dark brown
*V* = 1303.15 (8) Å^3^	0.30 × 0.06 × 0.04 mm
*Z* = 4	

### Data collection

**Table:**

Rigaku R-AXIS RAPID diffractometer	2430 reflections with *I* > 2σ(*I*)
graphite	*R*_int_ = 0.049
ω scans	θ_max_ = 27.5°, θ_min_ = 3.2°
Absorption correction: multi-scan (*ABSCOR*; Higashi, 1995)	*h* = −9→9
*T*_min_ = 0.602, *T*_max_ = 1.000	*k* = −16→14
12389 measured reflections	*l* = −17→17
2991 independent reflections	

### Refinement

**Table:**

Refinement on *F*^2^	133 parameters
Least-squares matrix: full	0 restraints
*R*[*F*^2^ > 2σ(*F*^2^)] = 0.034	*w* = 1/[σ^2^(*F*_o_^2^) + (0.0248*P*)^2^ + 3.4157*P*] where *P* = (*F*_o_^2^ + 2*F*_c_^2^)/3
*wR*(*F*^2^) = 0.075	(Δ/σ)_max_ < 0.001
*S* = 1.08	Δρ_max_ = 1.18 e Å^−^^3^
2991 reflections	Δρ_min_ = −1.27 e Å^−^^3^

### Special details

**Table:**

Geometry. All e.s.d.'s (except the e.s.d. in the dihedral angle between two l.s. planes) are estimated using the full covariance matrix. The cell e.s.d.'s are taken into account individually in the estimation of e.s.d.'s in distances, angles and torsion angles; correlations between e.s.d.'s in cell parameters are only used when they are defined by crystal symmetry. An approximate (isotropic) treatment of cell e.s.d.'s is used for estimating e.s.d.'s involving l.s. planes.

### Fractional atomic coordinates and isotropic or equivalent isotropic displacement parameters (Å^2^)

**Table:**

	*x*	*y*	*z*	*U*_iso_*/*U*_eq_	Occ. (<1)
Cs	−0.0009 (4)	0.02093 (15)	0.48715 (19)	0.0512 (5)	0.5
Ag	0	0	0	0.0539 (2)	
Nb1	0.42651 (5)	0.03565 (3)	0.24995 (3)	0.01333 (11)	
Nb2	0.43445 (5)	0.32590 (3)	0.25353 (3)	0.01280 (11)	
P	0.11427 (16)	0.18631 (9)	0.14370 (10)	0.0170 (3)	
S1	−0.03805 (19)	0.18878 (10)	0.02229 (11)	0.0292 (3)	
S2	0.07865 (16)	0.05281 (9)	0.22287 (10)	0.0210 (3)	
S3	0.08568 (16)	0.31790 (9)	0.22464 (10)	0.0207 (3)	
S4	0.33249 (16)	0.47121 (9)	0.35994 (9)	0.0187 (3)	
S5	0.38477 (17)	0.18131 (9)	0.37848 (9)	0.0190 (3)	
S6	0.39304 (16)	0.18214 (8)	0.11963 (9)	0.0161 (2)	
S7	0.42828 (17)	0.44324 (9)	0.10942 (9)	0.0194 (3)	
S8	0.58572 (16)	0.41695 (9)	0.39426 (9)	0.0184 (3)	
S9	0.63765 (16)	0.17797 (9)	0.31795 (9)	0.0189 (3)	
S10	0.67972 (16)	0.38737 (9)	0.14538 (9)	0.0204 (3)	

### Atomic displacement parameters (Å^2^)

**Table:**

	*U*^11^	*U*^22^	*U*^33^	*U*^12^	*U*^13^	*U*^23^
Cs	0.0270 (3)	0.0779 (16)	0.0489 (13)	−0.0003 (11)	0.0060 (7)	−0.0283 (10)
Ag	0.0518 (4)	0.0298 (3)	0.0800 (6)	−0.0028 (3)	0.0017 (4)	−0.0287 (4)
Nb1	0.0150 (2)	0.00814 (18)	0.0168 (2)	0.00081 (16)	−0.00185 (16)	0.00082 (16)
Nb2	0.0145 (2)	0.00802 (19)	0.0158 (2)	−0.00071 (16)	−0.00210 (16)	−0.00048 (16)
P	0.0157 (5)	0.0115 (5)	0.0235 (7)	0.0014 (5)	−0.0057 (5)	−0.0025 (5)
S1	0.0325 (7)	0.0228 (6)	0.0316 (8)	0.0023 (6)	−0.0168 (6)	−0.0038 (6)
S2	0.0181 (5)	0.0140 (5)	0.0310 (7)	−0.0014 (5)	−0.0023 (5)	0.0030 (5)
S3	0.0168 (5)	0.0146 (5)	0.0305 (7)	0.0023 (5)	−0.0034 (5)	−0.0073 (5)
S4	0.0190 (5)	0.0146 (5)	0.0225 (7)	−0.0007 (5)	0.0017 (5)	−0.0031 (5)
S5	0.0256 (6)	0.0126 (5)	0.0189 (6)	0.0001 (5)	0.0008 (5)	0.0007 (5)
S6	0.0186 (5)	0.0104 (5)	0.0193 (6)	0.0019 (5)	−0.0015 (5)	−0.0007 (5)
S7	0.0269 (6)	0.0131 (5)	0.0180 (6)	−0.0009 (5)	−0.0044 (5)	0.0004 (5)
S8	0.0233 (6)	0.0124 (5)	0.0195 (6)	−0.0013 (5)	−0.0043 (5)	0.0000 (5)
S9	0.0185 (5)	0.0128 (5)	0.0251 (7)	−0.0001 (5)	−0.0054 (5)	0.0009 (5)
S10	0.0217 (6)	0.0152 (5)	0.0244 (7)	−0.0003 (5)	0.0048 (5)	−0.0021 (5)

### Geometric parameters (Å, °)

**Table:**

Cs—Cs^i^	0.644 (3)	Nb2—S8	2.5075 (12)
Cs—S10^ii^	3.444 (3)	Nb2—S5	2.5643 (13)
Cs—S10^iii^	3.469 (3)	Nb2—S9	2.5670 (12)
Cs—S7^iv^	3.536 (3)	Nb2—S3	2.5920 (12)
Cs—S7^v^	3.581 (3)	Nb2—S6	2.6250 (12)
Cs—S2	3.722 (3)	Nb2—Nb1^viii^	2.8843 (5)
Cs—S1^v^	3.773 (2)	P—S1	1.9962 (18)
Cs—S5	3.835 (3)	P—S3	2.0391 (17)
Cs—S3^v^	3.915 (3)	P—S2	2.0527 (17)
Cs—S3^iv^	3.956 (3)	P—S6	2.0851 (17)
Cs—S9^vi^	4.044 (3)	S1—Cs^ix^	3.773 (2)
Cs—S2^i^	4.157 (3)	S2—Cs^i^	4.157 (3)
Ag—S1	2.4625 (13)	S3—Cs^ix^	3.915 (3)
Ag—S1^vii^	2.4625 (13)	S3—Cs^x^	3.956 (3)
Nb1—S4^iii^	2.4953 (13)	S4—S8	2.0371 (17)
Nb1—S7^iii^	2.4958 (12)	S4—Nb1^viii^	2.4953 (13)
Nb1—S8^iii^	2.5055 (13)	S5—S9	2.0542 (18)
Nb1—S10^iii^	2.5231 (13)	S7—S10	2.0372 (17)
Nb1—S9	2.5658 (12)	S7—Nb1^viii^	2.4958 (12)
Nb1—S2	2.5895 (12)	S7—Cs^x^	3.536 (3)
Nb1—S5	2.5993 (13)	S7—Cs^ix^	3.581 (3)
Nb1—S6	2.6103 (12)	S8—Nb1^viii^	2.5055 (13)
Nb1—Nb2^iii^	2.8843 (5)	S9—Cs^xi^	4.044 (3)
Nb2—S10	2.4910 (13)	S10—Nb1^viii^	2.5231 (13)
Nb2—S7	2.4932 (13)	S10—Cs^xii^	3.444 (3)
Nb2—S4	2.4985 (12)	S10—Cs^viii^	3.469 (3)
			
Cs^i^—Cs—S10^ii^	86.8 (5)	S9—Nb1—Nb2^iii^	117.38 (3)
Cs^i^—Cs—S10^iii^	82.5 (5)	S2—Nb1—Nb2^iii^	115.30 (3)
S10^ii^—Cs—S10^iii^	169.32 (5)	S5—Nb1—Nb2^iii^	136.98 (3)
Cs^i^—Cs—S7^iv^	88.8 (5)	S6—Nb1—Nb2^iii^	133.76 (3)
S10^ii^—Cs—S7^iv^	73.85 (7)	S10—Nb2—S7	48.25 (4)
S10^iii^—Cs—S7^iv^	105.80 (8)	S10—Nb2—S4	110.06 (4)
Cs^i^—Cs—S7^v^	80.9 (5)	S7—Nb2—S4	90.81 (4)
S10^ii^—Cs—S7^v^	105.35 (8)	S10—Nb2—S8	89.91 (4)
S10^iii^—Cs—S7^v^	73.00 (7)	S7—Nb2—S8	109.55 (4)
S7^iv^—Cs—S7^v^	169.64 (5)	S4—Nb2—S8	48.02 (4)
Cs^i^—Cs—S2	128.9 (5)	S10—Nb2—S5	138.22 (4)
S10^ii^—Cs—S2	134.71 (8)	S7—Nb2—S5	166.52 (4)
S10^iii^—Cs—S2	54.41 (5)	S4—Nb2—S5	95.72 (4)
S7^iv^—Cs—S2	79.52 (6)	S8—Nb2—S5	83.45 (4)
S7^v^—Cs—S2	107.01 (8)	S10—Nb2—S9	91.02 (4)
Cs^i^—Cs—S1^v^	139.1 (6)	S7—Nb2—S9	136.48 (4)
S10^ii^—Cs—S1^v^	61.86 (5)	S4—Nb2—S9	122.02 (4)
S10^iii^—Cs—S1^v^	127.50 (7)	S8—Nb2—S9	80.28 (4)
S7^iv^—Cs—S1^v^	105.15 (7)	S5—Nb2—S9	47.20 (4)
S7^v^—Cs—S1^v^	82.99 (6)	S10—Nb2—S3	130.35 (5)
S2—Cs—S1^v^	91.71 (4)	S7—Nb2—S3	84.19 (4)
Cs^i^—Cs—S5	131.1 (6)	S4—Nb2—S3	79.18 (4)
S10^ii^—Cs—S5	125.61 (7)	S8—Nb2—S3	124.13 (4)
S10^iii^—Cs—S5	62.86 (5)	S5—Nb2—S3	85.47 (4)
S7^iv^—Cs—S5	131.64 (7)	S9—Nb2—S3	126.33 (4)
S7^v^—Cs—S5	57.45 (5)	S10—Nb2—S6	83.03 (4)
S2—Cs—S5	55.08 (4)	S7—Nb2—S6	82.29 (4)
S1^v^—Cs—S5	64.82 (4)	S4—Nb2—S6	155.03 (4)
Cs^i^—Cs—S3^v^	88.9 (5)	S8—Nb2—S6	156.36 (4)
S10^ii^—Cs—S3^v^	52.55 (5)	S5—Nb2—S6	86.88 (4)
S10^iii^—Cs—S3^v^	126.89 (9)	S9—Nb2—S6	77.33 (4)
S7^iv^—Cs—S3^v^	126.40 (9)	S3—Nb2—S6	76.27 (4)
S7^v^—Cs—S3^v^	53.89 (5)	S10—Nb2—Nb1^viii^	55.41 (3)
S2—Cs—S3^v^	137.18 (6)	S7—Nb2—Nb1^viii^	54.72 (3)
S1^v^—Cs—S3^v^	51.72 (4)	S4—Nb2—Nb1^viii^	54.67 (3)
S5—Cs—S3^v^	86.11 (6)	S8—Nb2—Nb1^viii^	54.84 (3)
Cs^i^—Cs—S3^iv^	81.7 (5)	S5—Nb2—Nb1^viii^	138.05 (3)
S10^ii^—Cs—S3^iv^	126.35 (9)	S9—Nb2—Nb1^viii^	119.58 (3)
S10^iii^—Cs—S3^iv^	52.02 (5)	S3—Nb2—Nb1^viii^	112.65 (3)
S7^iv^—Cs—S3^iv^	53.79 (5)	S6—Nb2—Nb1^viii^	133.06 (3)
S7^v^—Cs—S3^iv^	123.87 (8)	S1—P—S3	112.52 (8)
S2—Cs—S3^iv^	51.42 (5)	S1—P—S2	112.54 (8)
S1^v^—Cs—S3^iv^	137.41 (7)	S3—P—S2	112.78 (8)
S5—Cs—S3^iv^	100.03 (7)	S1—P—S6	113.93 (9)
S3^v^—Cs—S3^iv^	170.63 (4)	S3—P—S6	102.73 (7)
Cs^i^—Cs—S9^vi^	137.7 (6)	S2—P—S6	101.48 (7)
S10^ii^—Cs—S9^vi^	75.21 (6)	P—S1—Ag	91.46 (6)
S10^iii^—Cs—S9^vi^	113.01 (7)	P—S1—Cs^ix^	94.72 (7)
S7^iv^—Cs—S9^vi^	49.70 (4)	Ag—S1—Cs^ix^	161.76 (7)
S7^v^—Cs—S9^vi^	140.51 (6)	P—S2—Nb1	90.68 (5)
S2—Cs—S9^vi^	59.66 (4)	P—S2—Cs	129.50 (7)
S1^v^—Cs—S9^vi^	62.08 (4)	Nb1—S2—Cs	91.32 (6)
S5—Cs—S9^vi^	89.44 (4)	P—S2—Cs^i^	136.28 (7)
S3^v^—Cs—S9^vi^	108.21 (6)	Nb1—S2—Cs^i^	89.76 (5)
S3^iv^—Cs—S9^vi^	79.11 (6)	P—S3—Nb2	90.27 (5)
Cs^i^—Cs—S2^i^	44.2 (5)	P—S3—Cs^ix^	89.92 (6)
S10^ii^—Cs—S2^i^	50.32 (4)	Nb2—S3—Cs^ix^	104.61 (6)
S10^iii^—Cs—S2^i^	120.06 (6)	P—S3—Cs^x^	99.22 (6)
S7^iv^—Cs—S2^i^	99.18 (6)	Nb2—S3—Cs^x^	103.24 (6)
S7^v^—Cs—S2^i^	73.35 (5)	S8—S4—Nb1^viii^	66.22 (5)
S2—Cs—S2^i^	173.07 (6)	S8—S4—Nb2	66.22 (5)
S1^v^—Cs—S2^i^	95.19 (7)	Nb1^viii^—S4—Nb2	70.56 (3)
S5—Cs—S2^i^	127.91 (8)	S9—S5—Nb2	66.47 (5)
S3^v^—Cs—S2^i^	48.73 (4)	S9—S5—Nb1	65.71 (5)
S3^iv^—Cs—S2^i^	122.41 (5)	Nb2—S5—Nb1	92.55 (4)
S9^vi^—Cs—S2^i^	124.54 (8)	S9—S5—Cs	146.11 (7)
S1—Ag—S1^vii^	180.00 (10)	Nb2—S5—Cs	140.15 (6)
S4^iii^—Nb1—S7^iii^	90.82 (4)	Nb1—S5—Cs	88.67 (5)
S4^iii^—Nb1—S8^iii^	48.08 (4)	P—S6—Nb1	89.39 (5)
S7^iii^—Nb1—S8^iii^	109.54 (4)	P—S6—Nb2	88.37 (5)
S4^iii^—Nb1—S10^iii^	109.13 (4)	Nb1—S6—Nb2	90.92 (4)
S7^iii^—Nb1—S10^iii^	47.89 (4)	S10—S7—Nb2	65.82 (5)
S8^iii^—Nb1—S10^iii^	89.23 (4)	S10—S7—Nb1^viii^	66.76 (5)
S4^iii^—Nb1—S9	91.47 (4)	Nb2—S7—Nb1^viii^	70.64 (3)
S7^iii^—Nb1—S9	78.95 (4)	S10—S7—Cs^x^	171.29 (7)
S8^iii^—Nb1—S9	137.40 (4)	Nb2—S7—Cs^x^	118.24 (6)
S10^iii^—Nb1—S9	121.46 (4)	Nb1^viii^—S7—Cs^x^	121.50 (5)
S4^iii^—Nb1—S2	130.77 (4)	S10—S7—Cs^ix^	161.56 (7)
S7^iii^—Nb1—S2	124.06 (4)	Nb2—S7—Cs^ix^	117.07 (6)
S8^iii^—Nb1—S2	85.24 (4)	Nb1^viii^—S7—Cs^ix^	131.67 (5)
S10^iii^—Nb1—S2	80.24 (4)	S4—S8—Nb1^viii^	65.70 (5)
S9—Nb1—S2	125.60 (4)	S4—S8—Nb2	65.76 (5)
S4^iii^—Nb1—S5	138.34 (4)	Nb1^viii^—S8—Nb2	70.25 (3)
S7^iii^—Nb1—S5	82.43 (4)	S5—S9—Nb1	67.42 (5)
S8^iii^—Nb1—S5	167.42 (4)	S5—S9—Nb2	66.33 (5)
S10^iii^—Nb1—S5	96.47 (4)	Nb1—S9—Nb2	93.27 (4)
S9—Nb1—S5	46.87 (4)	S5—S9—Cs^xi^	111.56 (7)
S2—Nb1—S5	84.69 (4)	Nb1—S9—Cs^xi^	104.00 (5)
S4^iii^—Nb1—S6	83.16 (4)	Nb2—S9—Cs^xi^	160.34 (6)
S7^iii^—Nb1—S6	155.62 (4)	S7—S10—Nb2	65.93 (5)
S8^iii^—Nb1—S6	83.80 (4)	S7—S10—Nb1^viii^	65.35 (5)
S10^iii^—Nb1—S6	155.76 (4)	Nb2—S10—Nb1^viii^	70.23 (3)
S9—Nb1—S6	77.61 (4)	S7—S10—Cs^xii^	110.68 (8)
S2—Nb1—S6	76.07 (4)	Nb2—S10—Cs^xii^	176.58 (7)
S5—Nb1—S6	86.46 (4)	Nb1^viii^—S10—Cs^xii^	109.02 (5)
S4^iii^—Nb1—Nb2^iii^	54.77 (3)	S7—S10—Cs^viii^	108.80 (8)
S7^iii^—Nb1—Nb2^iii^	54.64 (3)	Nb2—S10—Cs^viii^	168.71 (5)
S8^iii^—Nb1—Nb2^iii^	54.91 (3)	Nb1^viii^—S10—Cs^viii^	98.55 (4)
S10^iii^—Nb1—Nb2^iii^	54.37 (3)		

Symmetry codes: (i) −*x*, −*y*, −*z*+1; (ii) *x*−1, −*y*+1/2, *z*+1/2; (iii) −*x*+1, *y*−1/2, −*z*+1/2; (iv) −*x*, *y*−1/2, −*z*+1/2; (v) *x*, −*y*+1/2, *z*+1/2; (vi) *x*−1, *y*, *z*; (vii) −*x*, −*y*, −*z*; (viii) −*x*+1, *y*+1/2, −*z*+1/2; (ix) *x*, −*y*+1/2, *z*−1/2; (x) −*x*, *y*+1/2, −*z*+1/2; (xi) *x*+1, *y*, *z*; (xii) *x*+1, −*y*+1/2, *z*−1/2.
